# Biological Effectiveness of Accelerated Protons for Chromosome Exchanges

**DOI:** 10.3389/fonc.2015.00226

**Published:** 2015-10-19

**Authors:** Kerry A. George, Megumi Hada, Francis A. Cucinotta

**Affiliations:** ^1^Wyle Science, Technology and Engineering Group, Houston, TX, USA; ^2^University of Nevada Las Vegas, Las Vegas, NV, USA

**Keywords:** chromosomal aberrations, biomarkers, protons, proton therapy, space radiation

## Abstract

We have investigated chromosome exchanges induced in human cells by seven different energies of protons (5–2500 MeV) with LET values ranging from 0.2 to 8 keV/μm. Human lymphocytes were irradiated *in vitro* and chromosome damage was assessed using three-color fluorescence *in situ* hybridization chromosome painting in chemically condensed chromosomes collected during the first cell division post irradiation. The relative biological effectiveness (RBE) was calculated from the initial slope of the dose–response curve for chromosome exchanges with respect to low dose and low dose-rate γ-rays (denoted as RBE_max)_, and relative to acute doses of γ-rays (denoted as RBE_γAcute)_. The linear dose–response term was similar for all energies of protons, suggesting that the decrease in LET with increasing proton energy was balanced by the increase in dose from the production of nuclear secondaries. Secondary particles increase slowly above energies of a few hundred megaelectronvolts. Additional studies of 50 g/cm^2^ aluminum shielded high-energy proton beams showed minor differences compared to the unshielded protons and lower RBE values found for shielded in comparison to unshielded beams of 2 or 2.5 GeV. All energies of protons produced a much higher percentage of complex-type chromosome exchanges when compared to acute doses of γ-rays. The implications of these results for space radiation protection and proton therapy are discussed.

## Introduction

The study of the biological effectiveness of accelerated proton exposures is of interest for clinical treatment plans and for assessing normal tissue damage from protons of various energies that are generated outside of the Bragg peak during proton therapy (1–4). Protons are also a concern for space radiation exposures to astronauts because the space radiation flux is predominantly energetic protons or secondary protons produced in nuclear interactions (5–7). Although evidence now indicates that relative biological effectiveness (RBE) varies considerably along the proton depth-dose distribution, RBE modeling in treatment planning still involves significant uncertainties and, consequently, clinical proton therapy is usually based on the use of a generic RBE of 1.1 (4). Further experimental data are required before a consensus can be reached on weighting factors across the depth-dose profile and for different tissue effects.

Experimental studies have shown that the RBE of protons varies with biological endpoint, tissue type, dose, and energy of the protons. RBE values calculated by cell killing and mutation induction indicate that low energy protons are significantly higher than unity and values are LET dependent (8). Published data on chromosome damage have indicated that RBEs or RBE_max_ values for protons of energies above 10 MeV vary from <1 to about 2 in comparison to X-rays or γ-rays, whereas lower energy protons (<10 MeV) were significantly higher than unity and the values were LET dependent (9–11). RBEs for tumor induction were close to 1 in several studies (12, 13), and as high as 2 for Harderian gland tumors (14) and for rat mammary carcinomas (15) that were induced by 250 MeV protons. The choice of reference radiation can complicate the analysis of RBE because differences have been found for X-rays and γ-rays (16), and variability has been reported for low doses of photons and protons. In addition, high-energy protons induce nuclear spallation and other interactions that produce secondary protons, neutrons, and heavy ion fragments. Nuclear interaction cross sections generally increase with the energy of the protons (3), and the secondary particles typically have higher LET values that can increase RBE.

In the present study, we considered the induction of simple and complex-type chromosome exchanges in normal human lymphocytes. Chromosome exchanges, especially translocations, are positively correlated with many cancers, and are therefore a potential biomarker of cancer risk associated with radiation exposure (17–19). In addition, RBE factors for chromosome aberrations are similar to RBEs observed for induction of solid tumors in mice (16, 20, 21). Therefore, chromosome exchanges are a useful biomarker for cancer risk and can be compared with other biomarkers in the absence of human data for galactic cosmic rays (GCR). In earlier work (22), we considered the effects of 250 MeV protons at different dose-rates. Here, we consider several proton energies from 5 to 2500 MeV with additional studies on the effects of heavy aluminum and polyethylene shielding for the high-energy proton exposures.

## Materials and Methods

These studies were conducted in accordance with accepted ethical and humane practices, and were approved by the appropriate institutional and/or governmental committee(s) and/or organization(s).

### Irradiation

Whole blood was collected from healthy volunteers and was irradiated with accelerated protons using the NASA Space Radiation Laboratory (NSRL) facility at Brookhaven National Laboratory (BNL). The same volunteer donated the blood samples for each experiment. All samples were exposed in the plateau portion of the Bragg curves and dose rates were between 0.2 and 0.5 Gy/min, depending on the dose delivered. Doses were measured at the target using ionization chambers. Samples were exposed at room temperature. Each sample received at least three pulses and no exposure lasted more than 10 min. The beam uniformity was checked using a digital beam imager and dose did not vary more than 5% over the target area. For the 2.5 and 2 GeV protons exposures, the target areas was shielded, respectively, with 50 g/cm^2^ of aluminum and 50 g/cm^2^ of aluminum plus 10 cm of polyethylene. At these proton energies, the dose increases as the protons pass through the shielding due to secondary radiation, and doses were normalized using BNL dosimetry to generate the same total dose to the sample as the unshielded studies.

### Cell Culture

Immediately after exposure, whole-blood cultures in RPMI 1640 medium (Gibco BRL) supplemented with 20% calf serum and 1% phytohemagglutinin (Gibco, BRL) were incubated at 37°C for 48–50 h. Chemically induced PPC were collected using the method described by Durante et al. (23), which results in well-condensed chromosomes from cells in G2 and metaphase. Briefly, 50 nM calyculin A (Wako Chemicals) was added to the growth medium for the last 30 min of the incubation. Cells were then treated with hypotonic KCl (0.075M) for 15 min at 37°C and fixed in methanol:acetic acid (3:1). A 0.5 ml volume of blood from each sample was cultured with 10 μm bromodeoxyuridine (BrdU), and a differential replication staining procedure was completed on chromosomes from these samples by incubating slides in 0.5 mg/ml of Hoechst during exposure to black light (General Electric 15T8/BL bulb). Chromosomes were stained with Giemsa to visualized replication rounds, revealing the percentage of cells in first mitosis was >95% for all samples analyzed.

### Fluorescence *In situ* Hybridization

Chromosomes were dropped onto clean microscope slides and hybridized *in situ* with a combination of fluorescence whole-chromosome probes for chromosomes 1, 2, and 4, or chromosome 1, 2, and 5 (Rainbow Scientific) using the procedures recommended by the manufacturer. Chromosome 1 was painted with a Texas red fluorophore, chromosome 2 was painted with FTIC, and chromosome 4 (or 5) was painted with a 1:1 combination of Texas Red and FITC that appeared yellow under the triple-band-pass filter set. Unlabeled chromosomes were always counterstained with 4′,6-diamidino-2-phenylindole (DAPI).

### Chromosome Analysis

Chromosomes were analyzed on a Zeiss Axioplan fluorescence microscope. The images of all damaged cells were captured electronically using a Sensys charge-coupled device (CCD) camera (Photometrics Ltd., AZ, USA) and the Cytovision computer software. The number of cells analyzed for each sample varied, exact numbers are listed in Table 1. All slides analyzed in this study were coded and scored blind. Complex exchanges were scored when it was determined that an exchange involved a minimum of three breaks in two or more chromosomes (24). An exchange was defined as simple if it appeared to involve two breaks in two chromosomes, that is, dicentrics and translocations. Incomplete translocations and incomplete dicentrics were included in the category of simple exchanges, assuming that in most cases the reciprocal fragments were below the level of detection (25). Each type of exchange – dicentrics, apparently simple reciprocal exchanges, incompletes, or complex exchanges – was counted as one exchange, and values for total exchanges were derived by adding the yields. When two or more painted chromosomes were damaged, each was scored separately.

**Table 1:** **Dose–response data for chromosome aberrations per 100 cells induced by 5 different energies of protons measured in first post irradiation chemically induced PCC**.

Dose (Gy)	Cells scored	Simple exchanges	Complex exchanges
**E **=** 5 MeV**			
0.10	1018	2.3 ± 0.7	0 ± 0
0.20	1044	1.2 ± 0.9	0.7 ± 0.4
0.40	909	6.8 ± 1.6	1.1 ± 0.6
0.70	869	8.0 ± 1.7	2.1 ± 0.8
1.00	634	14.9 ± 2.6	4.3 ± 1.3
**E **=** 120 MeV^a^**			
0.15	1188	1.5 ± 0.6	0.4 ± 0.3
0.30	1437	2.1 ± 0.6	1.0 ± 0.4
0.50	1369	3.6 ± 0.8	2.7 ± 0.7
0.75	1136	6.1 ± 1.2	2.4 ± 0.7
1.00	825	13.8 ± 2.0	3.6 ± 1.0
1.50	357	31.2 ± 4.7	11.1 ± 2.8
2.00	203	61.0 ± 8.6	34.2 ± 6.5
**E **=** 250 MeV**			
0.25	491	1.9 ± 1.8	3.1 ± 1.3
0.50	536	2.8 ± 1.8	5.3 ± 1.6
0.80	427	5.2 ± 2.3	3.6 ± 1.5
1.20	563	7.6 ± 2.3	5.5 ± 1.6
2.00	325	29.4 ± 5.1	11.1 ± 3.0
**E **=** 800 MeV**			
0.25	330	0 ± 1.1	2.3 ± 1.4
0.50	609	0 ± 0.8	0.4 ± 0.4
0.80	655	13.8 ± 2.6	5.1 ± 1.4
1.20	561	14.0 ± 2.9	6.0 ± 1.7
2.00	263	35.2 ± 6.2	7.8 ± 2.8
**E **=** 1000 MeV**			
0.20	231	3.0 ± 2.2	0 ± 0
1.20	321	13.0 ± 3.7	4.3 ± 2.2
3.00	134	87.9 ± 15.1	38.8 ± 10.0
**E **=** 2000 MeV**			
0.25	330	0.7 ± 1.3	0.8 ± 0.8
0.50	284	9.7 ± 3.2	6.1 ± 2.3
0.80	378	13.5 ± 3.1	3.3 ± 1.5
1.20	538	9.9 ± 2.3	7.4 ± 1.8
2.00	243	46.3 ± 7.0	15.3 ± 4.0
**E **=** 2500 MeV**			
0.20	1342	1.4 ± 0.5	0.8 ± 0.4
0.40	1127	3.4 ± 0.9	2.1 ± 0.7
0.60	1635	7.6 ± 1.1	2.6 ± 0.6
0.80	218	7.1 ± 2.9	4.7 ± 2.4
1.20	304	24.7 ± 4.6	4.3 ± 1.9

*Data represent whole-genome equivalent values with background subtracted. ^a^150 MeV protons with 5 cm polyethylene shielding leading to residual energy of 120 MeV*.

### Statistical Analysis

The frequency of chromosomal aberrations in the painted chromosomes was evaluated as the ratio between aberrations scored and total cells analyzed. Several studies have indicated that the distribution of radiation damage among chromosomes is random, and the yield of exchanges measured within the first division after exposure is proportional to the DNA content of the chromosome analyzed, with some fluctuation of data (26). Therefore, the frequencies of exchanges in individual chromosomes can be extrapolated to whole-genome equivalents using a modified version of the Lucas et al. (27) formula, Fp = 2.05[fp(1−fp) + fp1fp2 + fp1fp3 + fp2fp3]FG. Fp is the combined frequency of exchanges in all painted chromosomes, fp is the fraction of the whole genome comprised of the painted chromosomes, fp1, fp2, and fp3 are the fractions of the genome for each individual chromosome, and FG is the whole-genome aberration frequency. Using this formula, the genomic frequency for a male donor was estimated as 2.48 times that detected in chromosomes 1, 2, and 4.

Standard errors for aberration frequencies were calculated assuming Poisson statistics. Error bars in each figure represent SEs of the mean values. The data were modeled assuming binomial errors per number of chromosomes analyzed with the frequencies of aberrations of various types extrapolated to whole-genome equivalents as described above.

A weighted linear-quadratic (LQ) or linear (L) regression model was used to fit dose–responses for each proton energy, and the γ-ray dose–responses. Using the maximum likelihood method, the linear and quadratic coefficients α and β in

$$Y=Y_{0}+\alpha D+\beta D^{2}$$

were found for simple, complex, and total exchanges. Estimates of RBE were made from the α-coefficient from the acute response (21), denoted as RBE_γAcute_, and from the ratio of initial slopes for γ-rays using our previous data (28–30) of low dose and low dose-rate irradiation, denoted as RBE_max_. For estimating a low dose and low dose-rate γ-ray component, we combined the data from our previous analysis of 0.1 Gy/h with additional data at low doses (<0.5 Gy) from the same volunteer used for the proton experiments. For complex exchanges, the low dose and dose-rate γ-rays, complex exchanges were rare and RBE_max_ estimates could not be made.

## Results

**Figure 1:**
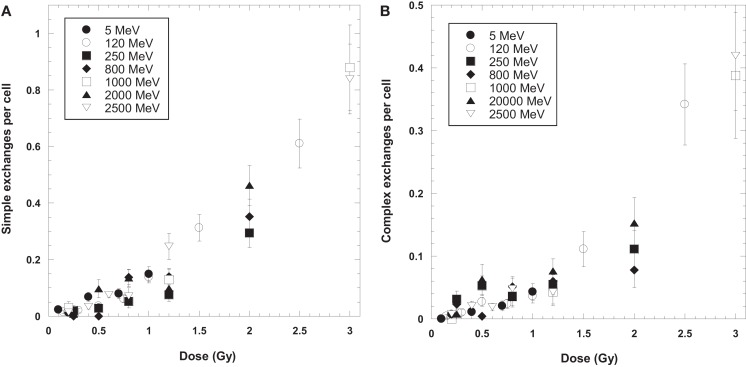
**Dose response curves for simple (A) and complex (B) chromosome exchanges induced by each ion**. Error bars indicate SEMs and background values have been subtracted for all data.

Tables 1 and 2 list the dose–response data for simple and complex-type chromosome exchanges for each energy of protons, and are represented as whole-genome equivalent values with background subtracted. The data, plotted in Figure 1, show a high degree of similarity in the dose–response for simple and complex exchanges for all proton energies considered. A weighted regression model based on the experimental errors was used to estimate α and β values with SEs for a linear-quadratic dose–response fit to the data for γ-rays and each proton energy. Tables 3–5 show results of this analysis for total exchanges, simple exchanges, and complex exchanges respectively. Comparison of the α values for acute and low dose rate (LDR) γ-rays fits indicates a dose-rate modifier factor of 1.83 and 1.74 for total exchanges and simple exchanges, respectively.

**Table 2:** **Dose–response data for chromosome exchanges per 100 cells induced by 2 and 2.5 GeV protons with and without shielding and measured in first post irradiation chemically induced PCC**.

Dose (Gy)	Cells scored	Simple exchanges	Complex exchanges
**E **=** 2000 MeV, no shielding**			
0.25	330	0.7 ± 1.3	0.8 ± 0.8
0.50	284	9.7 ± 3.2	6.1 ± 2.3
0.80	378	13.5 ± 3.1	3.3 ± 1.5
1.20	538	9.9 ± 2.3	7.4 ± 1.8
2.00	243	46.3 ± 7.0	15.3 ± 4.0
**E **=** 2000 MeV, 50 g/cm^2^ Aluminum **+** 10 cm polyethylene**			
0.25	401	1.3 ± 0.9	0.6 ± 0.6
0.5	1029	4.8 ± 1.1	2.0 ± 0.7
0.8	940	7.7 ± 1.5	1.6 ± 0.7
1.2	709	15.2 ± 2.4	4.4 ± 1.3
2.0	456	28.7 ± 4.0	3.0 ± 1.5
**E **=** 2500 MeV, no shielding**			
0.20	1342	1.4 ± 0.5	0.8 ± 0.4
0.40	1127	3.4 ± 0.9	2.1 ± 0.7
0.60	1635	7.6 ± 1.1	2.6 ± 0.6
0.80	218	7.1 ± 2.9	4.7 ± 2.4
1.20	304	24.7 ± 4.6	4.3 ± 1.9
**E **=** 2500 MeV, 50 g/cm^2^ aluminum**			
0.20	485	1.1 ± 0.8	0.5 ± 0.5
0.40	696	2.2 ± 0.9	0.7 ± 0.5
0.60	629	9.0 ± 1.9	2.5 ± 1.0
0.80	729	8.8 ± 1.8	3.5 ± 1.1
1.2	551	19.1 ± 3.0	9.3 ± 2.1

*Dose was measured at the target area for both shielded and unshielded exposures*.

*Data represent whole-genome equivalent values with background subtracted*.

**Table 3:** **Results for parameter estimates of linear-quadratic dose–response model for total exchanges, and relative biological effectiveness (RBE) factors for protons of different energies compared to acute, or low dose or low dose-rate **γ**-rays**.

Radiation type	α (Gy^−1^)	β (Gy^−2^)	RBE_γAcute_	RBE_max_
γ-Rays acute	0.176 ± 0.018	0.119 ± 0.038	–	–
γ-Rays LD	0.096 ± 0.01	–	–	–
Proton, 5 MeV	0.171 ± 0.018	0.043 ± 0.065	0.98 ± 0.11	1.78 ± 0.19
Proton, 120 MeV^a^	0.156 ± 0.016	0.167 ± 0.039	0.89 ± 0.09	1.62 ± 0.16
Proton, 250 MeV	0.144 ± 0.017	0.05 ± 0.032	0.82 ± 0.1	1.5 ± 0.18
Proton, 800 MeV	0.153 ± 0.036	0.114 ± 0.064	0.87 ± 0.21	1.59 ± 0.38
Proton, 1000 MeV	0.219 ± 0.037	0.12 ± 0.043	1.25 ± 0.21	2.27 ± 0.38
Proton, 2000 MeV	0.201 ± 0.033	0.093 ± 0.067	1.15 ± 0.19	2.09 ± 0.34
Proton, 2500 MeV	0.184 ± 0.006	0.105 ± 0.01	1.05 ± 0.05	1.91 ± 0.07

*^a^150 MeV protons with 5 cm polyethylene shielding leading to residual energy of 120 MeV*.

**Table 4:** **Results for parameter estimates of linear-quadratic dose–response model for simple exchanges, and relative biological effectiveness (RBE) factors for protons of different energies compared to acute or low dose or low dose-rate **γ**-rays**.

Radiation type	α (Gy^−1^)	β (Gy^−2^)	RBE_γAcute_	RBE_max_
γ-Rays acute	0.157 ± 0.013	0.092 ± 0.027	–	–
γ-Rays LD	0.09 ± 0.004	–	–	–
Proton, 5 MeV	0.132 ± 0.016	0.031 ± 0.057	0.84 ± 0.1	1.5 ± 0.18
Proton, 120 MeV^a^	0.121 ± 0.015	0.137 ± 0.036	0.77 ± 0.1	1.36 ± 0.17
Proton, 250 MeV	0.088 ± 0.009	0.064 ± 0.017	0.56 ± 0.06	1.0 ± 0.1
Proton, 800 MeV	0.116 ± 0.028	0.104 ± 0.049	0.73 ± 0.18	1.3 ± 0.31
Proton, 1000 MeV	0.159 ± 0.02	0.081 ± 0.023	1.01 ± 0.13	1.79 ± 0.23
Proton, 2000 MeV	0.132 ± 0.028	0.071 ± 0.058	0.84 ± 0.19	1.49 ± 0.31
Proton, 2500 MeV	0.119 ± 0.01	0.077 ± 0.015	0.76 ± 0.07	1.35 ± 0.11

*^a^150 MeV proton beam with 5 cm polyethylene shielding leading to residual energy of 120 MeV*.

**Table 5:** **Results for parameter estimates of linear-quadratic dose–response model for complex exchanges, and relative biological effectiveness (RBE) factors for protons of different energies compared to acute **γ**-rays**.

Radiation type	α (Gy^−1^)	β (Gy^−2^)	RBE_γAcute_
γ-Rays acute	0.015 ± 0.005	0.025 ± 0.014	–
Proton, 5 MeV	0.039 ± 0.004	0.006 ± 0.017	2.56 ± 0.85
Proton, 120 MeV^a^	0.032 ± 0.0043	0.024 ± 0.01	2.1 ± 0.28
Proton, 250 MeV	0.055 ± 0.009	0.03 ± 0.017	3.59 ± 1.3
Proton, 800 MeV	0.029 ± 0.010	0.02 ± 0.017	1.92 ± 0.87
Proton, 1000 MeV	0.06 ± 0.016	0.046 ± 0.022	3.96 ± 1.64
Proton, 2000 MeV	0.063 ± 0.009	0.028 ± 0.017	4.12 ± 1.41
Proton, 2500 MeV	0.058 ± 0.006	0.026 ± 0.01	3.81 ± 1.26

*RBE_max_ was not determined because low dose-rate γ-rays have very low induction of complex exchanges*.

*^a^150 MeV proton beam with 5 cm polyethylene shielding leading to residual energy at samples of 120 MeV*.

The linear (α) coefficients from the dose–response data (Tables 3–5) are similar for all energies as determined by either the LQ or L weighted regression models. The α values produced from the LQ models resulted in somewhat larger SD compared to fits from the linear weighted regression model (results not shown). RBE values for simple exchanges were slightly less or more than unity using the RBE_γAcute_ and RBE max models, respectively. However, a much higher frequency of complex exchanges was observed for each proton beam compared to γ-rays resulting in RBEs for complex exchanges varied from 2.1 to 4.1, and this led to a modest increase the RBEs for total exchanges. A trend toward increasing RBE_max_ values for proton energies of 1 GeV and higher was found for simple and total exchanges.

**Figure 2:**
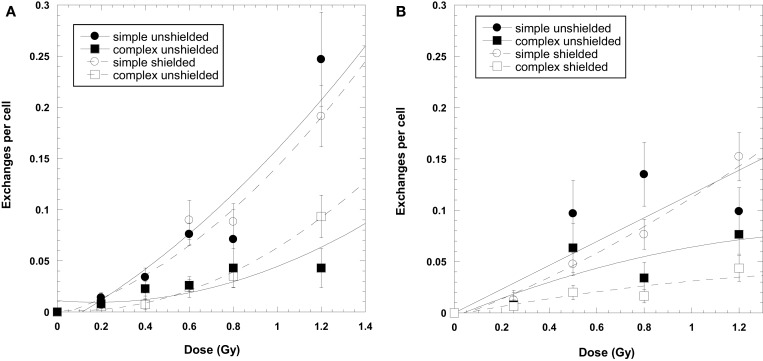
**Dose–response curves for chromosome exchanges induced by 2500 MeV protons (A) and 2000 MeV/μ protons (B)**. The 2000-MeV exposures were shielded with a combination of 50 g/cm^2^ aluminum and 10 cm polyethylene. The 2500-MeV exposure were shielded with 50 g/cm^2^ aluminum only. Error bars indicate SEMs and background values have been subtracted for all data points. Curve fit is extrapolated to the axis.

Data for the yield of chromosome exchanges in the shielded samples are listed in Table 2 where values are represented as whole-genome equivalent with background subtracted. The 2.5 GeV protons were shielded with 50 g/cm^2^ of aluminum, and the 2 GeV protons were shielded with 50 g/cm^2^ of aluminum plus 10 cm polyethylene. The doses represent the values measures at the target. A comparison of shielded and unshielded data shown in Figure 2 indicates similar dose–responses for the shielded and unshielded high-energy proton beams. However, RBE_max_ values were reduced with shielding. For example, RBE values for total exchanges induced by unshielded and shielded 2 GeV protons were 2.09 ± 0.34 and 1.26 ± 0.11, respectively, and values were 1.91 ± 0.67 and 1.53 ± 0.14 for unshielded and shielded 2.5 GeV protons, respectively.

## Discussion

The similarity in frequency of simple and complex exchanges over a wide range of proton energies found in our experiments suggests that decreases in LET with increasing proton energy is balanced by the increase in doses from secondary radiation, most notably secondary protons and neutrons (3, 31, 32). When the proton LET decreases from about 5 keV/μm at 5 MeV to 0.24 keV/μm at the highest energy of 2.5 GeV, there is concomitant increase in the contribution from nuclear secondaries and their contribution to the biological action cross section (3). Details of the beam characteristics for the shielded and unshielded protons used in our study are given in Table 6. Neutrons are produced in the absorbers or tissue equivalent materials through nuclear reactions by protons and other charged particles. Low energy neutrons (<5 MeV) are known to have large RBEs for different types of biological damage, including late effects (16). For our unshielded proton experiments, neutrons produced by the small amount of absorbing material present in the NSRL beam-line and biological samples themselves are largely high energy and unlikely to have slowed down to the more biologically effective neutron energies (<5 MeV). However, our experiments comparing shielding to unshielded protons at high energies led to similar yields of chromosome exchanges per unit dose. This is consistent with previous radiobiology studies with high-energy proton beams using very thick absorbers (33) and suggests that neutrons are ineffective in producing biological damage at high energy (>100 MeV). This observation is readily predicted by the mean-free path of neutrons which is generally >10 g/cm^2^ for materials of interest. Because the nuclear absorption cross sections are similar, secondary particles and target fragmentation spectrum produced by protons and neutrons are nearly identical for energies above a few hundred megaelectronvolts. Thus, high-energy protons are biologically more effective than neutrons of the same energy per unit fluence because of the proton charge state, while high-energy neutrons will have higher effectiveness per unit dose.

**Table 6:** **Details of protons shielding characteristics**.

Energy at beam entrance (MeV)	Shielding	Energy of beam at target (MeV)	LET (keV/**μ**m)	Percentage of dose from secondaries
5	None	–	7.80	–
120	5 cm polyethylene	42	0.64	6.4
250	None	220	0.43	17.4
800	None	770	0.24	33.1
1000	None	970	0.22	36.5
2000	None	1924	0.21	42.6
	50 g/cm^2^ aluminum + 10 cm polyethylene	1624	0.21	80.0
2500	None	2470	0.21	43.7
	50 g/cm^2^ aluminum	2241	0.21	78.4

*Shielding from booster window, ion chambers, and binary filter contribute 1.2 g/cm^2^ of aluminum equivalent shielding to all exposures (shielded or unshielded)*.

*LET reflects values for the beam only and does not include the effect of secondary particles*.

The NSRL beam-line and the sample holders provide a minimum of 1.2 g/cm^2^ of aluminum equivalent shielding. The additional shielding used in our experiments had a minor influence on the biological effectiveness when comparing unshielded high-energy proton beams because the secondary radiation produced behind the shielding will be of similar biological effectiveness as the primary beam, while similar numbers of low energy target fragments of high-LET produced for both the primary and secondary protons and neutrons will be produced for the shielded and unshielded beams.

The α values for acute and LDR γ-rays fits indicate a dose-rate modification factor of 1.83 and 1.74 for total exchanges and simple exchanges, respectively. These values are similar to those reported for dose-rate reduction factors found by Peng et al. (34), dose and dose-rate reduction effectiveness factors (DDREF) for tumor induction in mice (21), and larger than values reported for solid tumors in the atomic bomb survivors where a DDREF of 1.3 is estimated in the BEIR VII report (35).

In the present study, we used three-color combinations of fluorescence in situ hybridization (FISH) chromosome painting probes (chromosomes 1, 2, and 4, or 1, 2, and 5) to analyze our data. Presumably complex exchanges would be underestimated with this method due to the presence of some pseudosimple-type exchanges [that is, complex patterns that are indistinguishable from those created by simple reciprocal exchanges (36)]. Although the true complexity of exchanges can be determined only by analysis of all chromosomes, a significant number of chromosome exchanges found for all proton energies in this study were determined to be complex, which were significantly increased compared to acute or low dose-rate γ-rays.

In conclusion, our study of the proton energy dependence of chromosome exchanges in human lymphocytes suggests that biological effects are similar over a wide range of proton energies (5–2500 MeV) with RBE values for total exchanges are close to unity when measured against acute γ rays, and approach 2 when measured against low dose rate γ rays due to the increased number of complex exchanges at all proton energies compared to γ-rays.

## Conflict of Interest Statement

The authors declare that the research was conducted in the absence of any commercial or financial relationships that could be construed as a potential conflict of interest.
